# Correlation of Clinical Trachoma and Infection in Aboriginal Communities

**DOI:** 10.1371/journal.pntd.0000986

**Published:** 2011-03-15

**Authors:** Claude-Edouard C. Michel, Katrina G. Roper, Magda A. Divena, Helen H. Lee, Hugh R. Taylor

**Affiliations:** 1 Diagnostics Development Unit, Department of Haematology, University of Cambridge, National Health Service Blood and Transplant Site, Cambridge, United Kingdom; 2 Master of Applied Epidemiology Program, National Centre for Epidemiology and Population Health, College of Medicine and Health Sciences, Australian National University, Canberra, Australia; 3 Indigenous Eye Health Unit, Melbourne School of Population Health, University of Melbourne, Melbourne, Australia; 4 Vision CRC, Kensington, Australia; University of Cambridge, United Kingdom

## Abstract

**Background:**

Trachoma is the leading infectious cause of blindness due to conjunctival infection with *Chlamydia trachomatis*. The presence of active trachoma and evidence of infection are poorly correlated and a strong immunologically-mediated inflammatory response means that clinical signs last much longer than infection. This population-based study in five Aboriginal communities endemic for trachoma in northern Australia compared a fine grading of clinical trachoma with diagnostic positivity and organism load.

**Methods:**

A consensus fine grading of trachoma, based on clinical assessment and photograding, was compared to PCR, a lipopolysacharide (LPS)-based point-of-care (POC) and a 16S RNA-based nucleic acid amplification test (NAAT). Organism load was measured in PCR positive samples.

**Results:**

A total of 1282 residents, or 85.2% of the study population, was examined. Taking the findings of both eyes, the prevalence of trachomatous inflammation-follicular (TF) in children aged 1–9 years was 25.1% (96/383) of whom 13 (13.7%) were PCR positive on the left eye. When clinical data were limited to the left eye as this was tested for PCR, the prevalence of TF decreased to 21.4% (82/383). The 301 TF negative children, 13 (4.3%) were PCR positive. The fine grading of active trachoma strongly correlated with organism load and disease severity (rs = 0.498, *P* = 0.0004). Overall, 53% of clinical activity (TF_1_ or TF_2_) and 59% of PCR positivity was found in those with disease scores less than the WHO simplified grade of TF.

**Conclusion:**

Detailed studies of the pathogenesis, distribution and natural history of trachoma should use finer grading schemes for the more precise identification of clinical status. In low prevalence areas, the LPS-based POC test lacks the sensitivity to detect active ocular infection and nucleic acid amplification tests such as PCR or the 16S-RNA based NAAT performed better. Trachoma in the Aboriginal communities requires specific control measures.

## Author Summary

Repeated episodes of *C. trachomatis* infection lead to active trachoma clinically characterised by an often intense inflammatory response to chlamydial antigens with later scarring and distortion of the eyelid leading to blindness. However, the clinical signs of trachoma do not correlate well with laboratory tests to detect the presence of Chlamydia. The WHO simplified clinical grading scheme currently used for assessment of trachoma has a poor correlation with *C. trachomatis* genomic test findings, even though the detection of bacterial genome is strongly correlated with the prevalence and severity of active trachoma. A detailed assessment of the clinical signs using a finer grading system was studied in a population-based survey in five Australian Aboriginal communities. Much clinical activity and infection was found in those with clinical signs below the threshold used in the current WHO grading scheme. Future studies of the distribution of infection and pathogenesis should use finer grading methods than the current WHO scheme. The prevalence of trachoma in these communities confirms that trachoma remains of public health importance and sustained interventions to control trachoma are warranted.

## Introduction

Trachoma is the leading infectious cause of blindness [1], [2], [3], and results from repeated episodes of conjunctival infection by *Chlamydia trachomatis* (CT) serovars A, B, Ba and C. It is a major public health problem associated with poverty in environments with inadequate sanitation, poor personal hygiene and poor water supply and is now largely confined to developing countries, particularly in Sub-Saharan Africa [2], [4], [5], [6]. Nucleic acid amplification tests (NAATs) require appropriate facilities and skilled staff, but a assay designed for use in resource-limited settings may offer some advantages for the diagnosis of infection over clinical assessment [7], [8]. In general, irrespective of the diagnostic methodology, there is a relatively poor correlation between clinically active trachoma and biological evidence of infection, in part because signs of the disease are induced by a strong immunologically-mediated inflammatory response that resolves much more slowly than the infection [4], [5], [9], [10], [11], [12], [13]. It is further compounded by the occurrence of repeated episodes of infection. Also important is the relative lack of precision in assessing clinical status with the WHO simplified trachoma grading system [14], [15], which was designed to be learnt and used by local health workers and generally has a high level of reproducibility [16].

We sought to compare a fine consensus grading of trachoma combining clinical and photographic grading [15], [17] with a commercially available polymerase chain reaction (PCR), the CT/NG Amplicor test (Roche Diagnostic Corporation, IN, USA). We sought to compare field performance of a previously described POC assay [7] and a sensitive in-house 16S-RNA NAAT using an improved visual detection of nucleic acid by dipstick [18], [19] using the CT/NG Amplicor assay targeting one sequence coding for ORF1 (Open Reading Frame 1) of the *Chlamydia* cryptic plasmid as the reference test. Organism load was quantified with real-time quantitative PCR (qPCR) in CT positive individuals [20], [21].

## Methods

### Patients

Patients were recruited using the medical clinical list, the local council housing list and local knowledge of the Aboriginal Health Workers from five Aboriginal communities with endemic trachoma in the Katherine region of the Northern Territory Australia during a five week period over July and August 2007 as described previously [15], [17]. These communities had not received any recent azithromycin or other mass antibiotic treatment, although local health services do prescribe and dispense a range of broad-spectrum antibiotics on an individual patient basis that might indirectly have had an impact on trachoma and our findings. However, without access to confidential patient history, the recipients of these antibiotic prescriptions were not identifiable. In principle, school children in remote communities receive one annual health check that includes trachoma screening, but this process is patchy at best. Despite recommendations, very little screening for trichiasis in elders has been conducted in area where trachoma is endemic [15].

During the clinical assessment, both examiners wore two pairs of gloves. To limit the risk of cross-contamination between specimens and to prevent the exposure to transmittable diseases to consecutive subjects, utensils and surfaces [5], the outer pair was removed between successive participants and the inner pair disinfected and regularly disposed of. The examiner graded the clinical signs of trachoma using a fine grading scheme (Table 1) [15], [17]. Digital photographs were taken of the left inverted upper lid that were subsequently graded independently using the fine grading scheme and any discrepancies were adjudicated to give the final consensus grading [15], [17]. The clinical grading was also expressed in terms of the WHO simplified grading system as “Trachomatous inflammation-follicular” (referred to as TF_WHO_), “Trachomatous inflammation-intense” (TI_WHO_) and “Trachomatous scarring” (TS_WHO_) [14].

**Table 1:** Detailed grading scheme for clinical assessment of trachoma.

Grading	Definition of the finer grading
***TF - Trachomatous follicular:***	
TF_0_	No visible follicles in the upper tarsal conjunctiva
TF_1_	One or two small follicles in the upper tarsal conjunctiva
TF_2_	More than two but less than 5 follicles of 0.5 mm in diameter in the upper tarsal conjunctiva
TF_3_	Five or more follicles of 0.5 mm in diameter in the upper tarsal conjunctiva and equivalent to WHO simplified grading of TF
TF_4_	Extensive large follicles of 0.5 mm in diameter in the upper tarsal conjunctiva
***TI - Trachomatous inflammation – intense***	
TI_0_	No visible inflammation of the tarsal conjunctiva
TI_1_	Mild inflammation of the tarsal conjunctiva without obstruction of the vessels
TI_2_	Moderate inflammation of the tarsal conjunctiva with less than half of the deep tarsal vessels being obscured
TI_3_	Pronounced inflammatory thickening of the tarsal conjunctiva that obscures more than half of the normal deep tarsal vessels and equivalent to WHO simplified grading of TI
TI_4_	Very pronounced inflammation of the tarsal conjunctiva
***TS - Trachomatous scarring***	
TS_0_	No visible scarring of the tarsal conjunctiva
TS_1_	Small amount of early scarring apparent, but not clearly visible
TS_2_	Moderate amount of early scarring apparent, but not clearly visible
TS_3_	Presence of clearly visible scarring apparent in the upper tarsal conjunctiva and equivalent to WHO simplified grading of TS
TS_4_	Extensive clearly visible scarring involving most of the tarsal conjunctiva

After the clinical assessment and photography, two ocular swabs from the left eye were collected consecutively under stringent conditions to limit cross-contamination [5], and rigorous photographic cataloguing and sample labelling systems. Over 95% of samples were collected by the same swabber throughout the study to minimise any sampling variability. Additionally, gloves, surfaces, loupes, the camera and other utensils were swabbed twice a day to detect possible cross-contamination.

We obtained approval for the study from the Human Research Ethics Committees of the Royal Victorian Ear and Eye Hospital, the Australian National University, the Northern Australian National University and the Northern Territory Government Department of Health & Communities Services and Menzies School of Health Research. Signed written consent was obtained from each person, with consent for children under 18 years of age being provided by a parent or guardian [15], [17].

### Clinical examination

Clinical assessment can be difficult and inconsistent when conducted by poorly trained or inexperienced staff [5], [22]. Taking digital photographs, was previously described as an alternative method and compared to clinical assessment [15], [22]. Briefly, the majority of the examinations and taking digital photographs was done by examiner A (96%) to minimise inter-observer variability while the remaining examination were performed by examiner B who also examined and graded independently digital photographs without prior knowledge of the clinical assessment. In a masked fashion, both examiners re-examined photographs and gave an adjudicated score when either the clinical grade or the photographic grade was 3 or greater. A total of 88, 29 and 93 photographs were re-examined for the presence of follicles, inflammation and scarring, respectively. Weighted kappa analysis was previously reported to determine the concordance between methods. The data indicated that there was 79.7% agreement (kappa = 0.40) between clinical assessment, clinical grading and photographic assessment of trachomatous follicles (TF_1_-TF_4_) and 96.1% agreement (k = 0.71) when the fine score was translated to TF_WHO_. The agreement for TI_WHO_, and TS_WHO_ was 89.3% (k = 0.67) and 92.7% (k = 0.67), respectively [15]. Previous studies have shown the advantages of using finer scales to enhance the sensitivity of clinical measurement, although finer grading schemes may reduce the concordance, or frequency of perfect agreement, between the grades assigned by pairs of independent observations [23].

### Laboratory assays

The POC test was performed on site using the first left-eye ocular swab collected by only one experienced technician throughout the study. The assay detects chlamydial lipopolysaccharide (LPS) as previously described [7] with the following modifications for field use: 1) an alternative nitrocellulose membrane was used as the manufacturer discontinued the membrane previously used, 2) the ratio of lyophilised signal amplification system was modified for the test to function at high ambient temperature and 3) increased length of the conjugate tube which houses the dipstick to minimise the evaporation of the reagents during wicking and to protect the membrane against dust.

In addition to the lyophilised signal amplification reagents consisting of a biotin-labelled monoclonal antibody to chlamydial LPS and an anti-biotin monoclonal antibody conjugated to colloidal gold particles as colour indicator [7], the nitrocellulose-based membranes are the heart of lateral or vertical flow assays. The wicking rate, pore size, residual surfactants and detergents present on the matrix affect the characteristics of nitrocellulose-based membranes and reaction kinetics. Therefore, changing this porous substrate matrix and the addition of some features (i.e. shape of the conjugate tube) require a systematic adjustment of ratio of the lyophilized signal amplification reagents.

The anti-biotin monoclonal antibodies (clone BII-10A12A9A1, Diagnostic Development Unit, University of Cambridge, Cambridge, UK) conjugated to colloidal gold (British Biocell International, Cardiff, UK) by passive adsorption specifically bind to the lyophilised signal amplification reagents, consisting of a biotinylated monoclonal antibody to chlamydial LPS detection antibody (clone CTIII-10B9A10A4D28, Diagnostic Development Unit) biotinylated with the BAC-Sulfo-NHS-LC-biotin reagent (Sigma, St Louis, MO, USA) at a ratio of nine biotins per antibody molecule.

For LPS-POC testing, ocular swabs were placed in the sample extraction tube with a tapered bottom to facilitate extraction of the swab and a cap that allows it to also function as a dropper. The lysis reagent and analyte stabiliser were added sequentially as previously described [7]. Briefly, the lysis reagent (400 µL, Diagnostic Development Unit) and analyte stabiliser (300 µL, Diagnostic Development Unit) were added sequentially and mixed by gently dipping the swab to the bottom of the extraction tube three times after addition of each reagent. Two hundred microlitres of the above extract were immediately transferred to 800 µL of pre-dispensed Amplicor sample dilution buffer (Roche) for PCR testing. Thereafter, the signal enhancer reagent (33 µL, Diagnostic Development Unit) was added to each extract. This allows the release of chlamydial-LPS for detection. Five drops of the resulting extract (100 µL) were transferred to the detection tube into which the dipstick is placed. Two hundred microlitres of the above extract were immediately transferred to 800 µL of pre-dispensed Amplicor sample dilution buffer (Roche) for PCR testing. Thereafter, the signal enhancer reagent was added to each extract. This allows the release of chlamydial-LPS for detection. Five drops of the resulting extract were transferred to the detection tube into which the dipstick is placed. The detection tube contains lyophilised reagents of the signal amplification system consisting of biotinylated monoclonal antibody to chlamydial LPS and anti-biotin monoclonal antibodies conjugated to colloidal gold particles as the colour indicator. The dipstick contains a nitrocellulose membrane, lined with another monoclonal antibody to chlamydial-LPS (clone CVII-105A5A8, Diagnostic Development Unit) at the capture zone, which captures the immune complex formed between the chlamydial-LPS and signal amplification system reagents, if present. The accumulation of coloured conjugate at the capture line of the dipstick generates a visible colour change as previously described [7]. To generate a visual signal on the parallel to and above the capture zone, the dipstick was lined with the anti-biotin antibody described above, which served as the procedural control zone. All antibodies were produced in-house and purified by affinity chromatography to more than 95% purity before use.

For PCR testing, 200 µL of the POC extract, obtained before adding 6% H_2_O_2_, were mixed with 800 µL of Amplicor sample dilution buffer (Roche) and placed at 4 °C within 1 hr, and frozen at –20 °C within 2 days until transport to Cambridge, UK in dry-ice. These samples were stored at –80 °C until blind-tested by Amplicor.

The second matched swab was stored dry on cold packs, frozen at –20 °C within 2 days of collection and transported to Cambridge, UK in dry ice, and stored at –80 °C until tested to minimise any target degradation. For chlamydial and internal control testing, they were placed overnight in the Amplicor M4RT-transport medium (3 mL, Roche) and tested by one experienced technician according to the manufacturer's instruction (Roche).

All samples yielding a positive PCR result were quantified by previously described ethanol precipitation and qPCR methods [20], [21]. Briefly, homogenized M4RT-media (500 µL) from an Amplicor CT/NG Specimen Collection tube (Roche) containing the ocular swab were aliquoted into a DNase/RNase free siliconized tube (BioQuote, North Yorkshire, UK). Specimens were incubated at room temperature for 10 min prior to centrifugation at 17,860 *g* (max speed: 15,000 rpm) for 15 min at 25 °C (1.0R Megafuge). Supernatants obtained from diluted M4RT-media were decanted with sterile filter tips and the resulting pellets were re-suspended in 1 ml of cell culture grade Dulbecco's phosphate-buffered saline (DPBS) lacking Ca^2+^ and Mg^2+^ (BioWhittaker, Walkersville, MD) by vortexing. The re-suspended pellets were re-centrifuged as indicated above and re-suspended in 100 µL of 2M solution of ammonium hydroxide (obtained from a diluted 5N ammonium hydroxide volumetric standard, Sigma-Aldrich, St. Louis, MO, USA). Specimens were vortexed vigorously, incubated at room temperature for 10 minutes and vortexed again. If the pellet had not dissolved, it was solubilized by repeat pipetting and continuous cycle of vortexing until dissolved. Each specimen was placed into a heating block and heat-treated at 95–100 °C for 1 hour, or until the ammonia had evaporated (dry tubes). Dried specimens were re-suspended in 500 µL of molecular reagent-grade water and, vigorously vortexed and incubated at room temperature for ≥30 minutes to ensure that any precipitate had re-dissolved. The extracts were stored at 4 °C and tested within 24 h. The above extracted samples and standard curves were prepared and amplified in duplicate on two different days (4 data points) by Real-time qPCR.

Real-time qPCR was performed using a previously described method [20], [21] targeting one sequence coding for ORF1 of the *Chlamydia* cryptic plasmid [20], [21]. This method was previously demonstrated highly reproducible (R^2^ = 0.998) and with analytical sensitivity of <10 copies per amplification [21]. The previously described reproducibility was established against eleven standard curves constructed for the EB standard on different days. Each curve was generated from seven serial 10-fold dilutions of the pCTL12A plasmid amplified in duplicate. In addition, previously published data showed that 7.72±0.68 (mean ± SD) plasmid copies corresponded to one elementary body of *C. trachomatis* (serovar L1), consistent with previously obtained values [20]. Analysis of genital clinical specimens revealed a strong correlation (*R* ^2^ = 0.929) between elementary body counts determined by a quantitative ligase chain reaction (LCR)–based *Chlamydia trachomatis* LCx Assay (Abbott Laboratories) which targets a conserved region of the cryptic plasmid and those determined by the current qPCR method [20]. Although most of the infected patients were likely to harbour *C. trachomatis* serovars A, B, Ba and C, the primer sets for both Amplicor and qPCR assays correspond to conserved regions of the *C. trachomatis* cryptic plasmid and are therefore able to detect all *C. trachomatis* serovars. Through the present analysis, the organism load was expressed in number of plasmid per swab and not in EB per swab even though, to the knowledge of the authors, it has not been reported that the number of cryptic plasmid significantly varies between serovars. In addition, the second swab from patients identified as Amplicor-positive and 50 randomly selected Amplicor-negative samples with or without clinical signs were tested in duplicate with the 16S-RNA assay. The second swabs were tested in a masked fashion (randomised order) by Amplicor and the 16S-RNA assay. Sample that yielded a positive result on the first swab, but was negative on the second swab, was re-tested in a chessboard manner in presence of known positive and negative samples and, positive and negative controls.

Amplification of RNA extracted samples (total RNA RNeasy Mini Kit, QIAGEN Inc. Valencia, CA, USA) were performed by isothermal amplification and amplified products detected visually on a dipstick as described previously [18], [19], [24], [25]. The test designated as SAMBA (Simple AMplification-Based Assay) is based on a proprietary technology [18], [19]. Primer and probe target conserved sequences for all the 16sRNA *Chlamydia trachomatis* serovars obtained from the American Type Culture Collection (ATCC; MD, USA). Regions are conserved for all *Chlamydia trachomatis* serovars and were selected as previously described for the diagnosis of 2009 pandemic influenza (H1N1) [25] with sequences obtained from the National Center for Biotechnology Information (http://www.ncbi.nlm.nih.gov) and analysed with Jalview 2.3 (University of Dundee, UK). Detector and capture probes [24] were also designed to target similarly these specific regions. The primers and probes were compared with the Nucleotide Collection database at NCBI with the use of the Basic Local Alignment Search Tool (BLAST). Specificity was established against a panel of microorganisms commonly associated with human eye and skin (e.g. Staphylococcus, Pseudomonas, Streptococcus, *Escherichia*, *Proteus* and Candida, obtained from ATCC). The SAMBA *Chlamydia* in a closed device to prevent amplicon contamination has the same unique characteristics of the previously described SAMBA HIV-1 test chemistry render it suitable for near-patient testing in both developed and developing countries because the test uses thermostable reagents and a simplified protocol with minimum sample processing [24].

In brief, after amplification, the amplicon was incubated in a 2 mL microcentrifuge tube at 41 °C on a heating block. 20 µL of amplification product were added to a proprietary detection mixture and the dipstick was inserted in the reaction mixture. The test results were examined after 25 min of incubation and signal on the dipstick scored by an experienced operator according to the in-house scoring chart [25].

To assess the quality of the sampling procedure, human genomic DNA was quantified with Double-Dye Taqman kit according to the manufacturer's instruction (Primer Design, Southampton, UK) in all PCR positive samples and 41 randomly selected PCR-negative samples. The primers of human genomic DNA kit detect a single copy region of non-transcribed DNA.

### Statistical analysis

Statistical analysis was performed with SAS v9.1 software. Confidence intervals (CI) were calculated as exact binomials. The geometric mean of organism load as well as its respective standard deviation (SD) and 95% confidence interval (CI) for the ocular swabs were calculated from the natural log transformation of the organism load obtained for each swab. The organism load of the ocular samples was compared between the first and second grades of the clinical signs by the Student's *t*-test, unequal variance *t*-test Satterthwaite and equal variance pooled t-test. The correlation between organism load and the fine grading scheme, the load first swabs and the organism load of seconds swabs, and organism load between the different population was obtained using Spearman *Rho* (R) coefficients and paired Wilcoxon rank tests. Reliability of PCR positivity of both swabs or with 16S-RNA positivity was assessed with the kappa coefficient and its 95% confidence intervals. A *p*-value of <0.05 was considered statistically significant.

## Results

### Fine grading scheme of the clinical signs

**Figure 1:**
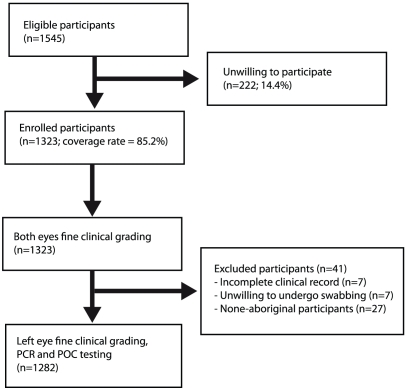
Recruitment algorithm for participants in five Aboriginal communities.

We examined 1316 of 1545 potential participants, giving an overall examination rate of 85.2% (Figure 1). A total of 1282 participants were eligible for this analysis with a median age of 17.1 years (range: 0.1–95). Each participant was assessed for clinical signs of trachoma using a fine grading scheme (Table 1, [15]), by PCR (Roche) and by the LPS-based POC assay.

On the basis of clinical examination of both eyes of each subject, 135 participants had active trachoma (10.5%; 95% CI: 8.9–12.2), 130 with TF_WHO_ and five had TI_WHO_ without TF_WHO_. Taking the findings of both eyes the highest age-specific prevalence of TF was in children 2–4 year-old (33/121; 27.3%) followed by 5–9 year-old (51/229; 22.3%) and those with less than 2 years of age (13/61; 21.3%). The overall prevalence of TF_WHO_ in children aged 1–9 was 25.1% (96/383, 95% CI: 20.7–29.4) and the prevalence of the five communities ranged from 9.8% to 38.5% (4/41 [9.8%], 5/49 [10.2%], 23/115 [20.0%], 27/82 [32.9%] and 37/96 [38.5%], respectively).

In contrast, when clinical data were limited to the left eye in order to directly compare with the PCR and POC testing results, the frequency of clinical signs of active trachoma decreased to 8.6% (110/1282), 108 with TF_WHO_ and two had TI_WHO_ without TF_WHO_ (Table 2). The resulting prevalence in the five communities in children aged 1–9 was 0/41 (0%), 4/49 (8.2%), 21/115 (18.3%), 21/82 (25.6%) and 36/96 (37.5%).

**Table 2:** Clinical signs of active trachoma compared with the presence of *C. trachomatis.*

Clinical signs of active trachoma (TF and TI) in the left eye compared with the presence of *C. trachomatis* DNA by PCR and the POC test positivity							
Clinical sign	PCR Positive (*n* = 46)			POC Positive (*n* = 14)			Total
and photograding	*n*	%	95% CI^1^	*n*	%	95% CI^1^	(*n* = 1282)
**TF_0_**	6	0.8	0.3–1.7	0	0.0	0.0–0.4	793
**TF_1_**	16	6.2	3.8–9.9	2	0.8	0.0–3.0	257
**TF_2_**	5	4.0	1.5–9.3	1	0.8	0.0–4.9	124
**TF_3_**	16	16.3	10.2–25.0	9	9.2	4.7–16.7	98
**TF_4_**	3	30.0	10.3–60.8	2	20.0	4.6–52.1	10
**TF_WHO_ absent**	27	2.3	1.6–3.3	3	0.3	0.1–0.8	1,174
**TF_WHO_ present**	19	17.6	11.5–25.9	11	10.2	5.6–17.5	108^2^
**TF_WHO_ & TI_WHO_ present**	6	66.7	35.1–88.3	3	33.3	11.7–64.9	9
**TI_WHO_ present without TF_WHO_**	1	50.0	9.5–90.6	1	50.0	9.5–90.6	2
**Active trachoma**	20	18.2	12.0–26.5	12	10.9	6.2–18.3	110^3^

*^1^95% Confidence intervals were calculated with the adjusted Wald interval method.*

*^2^108 participants had TF_WHO_, 99 with TF_WHO_ and nine had TF_WHO_ with TI_WHO_*.

*^3^110 participants had active trachoma, 108 with TF_WHO_ and two had TI_WHO_ without TF_WHO_*.

### NAAT-positivity

**Figure 2:**
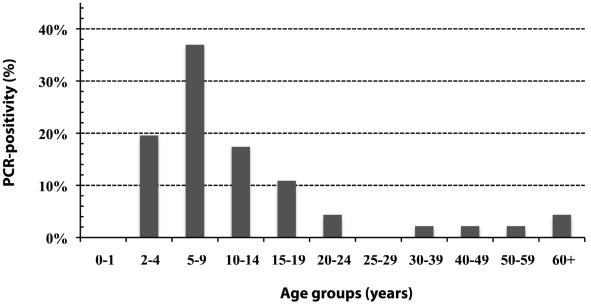
Age distribution of PCR-positive subjects (*n* = 46/1282).

The PCR (Amplicor) positivity rate in the population was 3.6% (46/1282, 95% CI: 2.6–4.6) and, in children aged 1–9, 6.8% (26/383, 95% CI: 4.3–9.3). Of the PCR positive participants, the highest rate was in children 5–9 year-old (17/46; 37%) followed by 2–4 year-old (9/46; 19.6%) and 10–14 year-old (8/46; 17.4%, Figure 2). Of the 46 people for whom the first swab from the left eye was PCR-positive, on testing of the second swab, 43 (93.5%, 95%CI: 86.3–100) were PCR-positive and 44 (95.7%) were 16S-RNA-positive. Two of the three PCR-negative second swabs were 16S-RNA-positive and one of those had the lowest organism load on the first swab. There was a good agreement between the first and second swabs tested with PCR (kappa coefficient 0.97; 95% CI: 0.93–1.00) and between the first swab PCR and the 16S-RNA result (kappa coefficient 0.98; 95% CI: 0.95–1.0).

**Figure 3:**
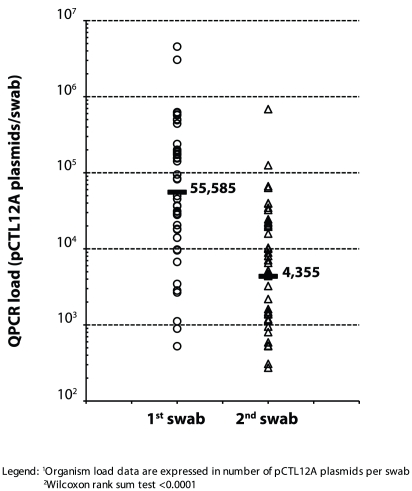
Distribution of the organism load^1^ in first and second swabs showing the geometric mean^2^.

The CT organism load was analysed by qPCR in all PCR-positive swabs (Figure 3). The geometric mean organism load was 55,585 (95%CI: 801–3,811,754) pCTL12A plasmid per swab for the first swab and 4,355 (95%CI: 98–193,602) for the second. The mean organism load for the second swab was 12.8 times lower (95%CI: 0.79–566.3) than for the first (paired Wilcoxon rank sum test, *P*<0.0001). The organism load in the first swab was strongly correlated with the load in the second swab (Spearman *Rho* = 0.74, *P*<0.0001).

To confirm the adequacy of specimen collection, human genomic DNA was quantified for the 46 PCR-positive (1.25±0.69 µg of genomic DNA/swab) and 41 random PCR-negative (including nine participants presenting signs of TF_WHO_ – 1.33±0.70 µg of genomic DNA/swab). The amount of genomic DNA was not significantly different between positive and negative ocular samples (two-tailed P = 0.6), nor was there a correlation between the organism load and the quantity of genomic DNA/swab. All of the 32 control swabs of potential formites were negative by PCR.

### NAAT-positivity versus clinical signs

A significant correlation was observed between PCR-positivity (Amplicor) and TF_WHO_ (Wilcoxon rank sum tests *P*<0.0001) and, between PCR-positivity and the fine grading scheme (Spearman *Rho* = 0.98 and *P* = 0.0004) (Table 2). A higher proportion of people were PCR positive as clinical disease, as assessed by the fine grading, became more severe. However, it should be noted that 59% (27/46) of PCR positive results occurred in people with TF_WHO_, although only 13% (6/46) occurred in people with TF_0_.

**Figure 4:**
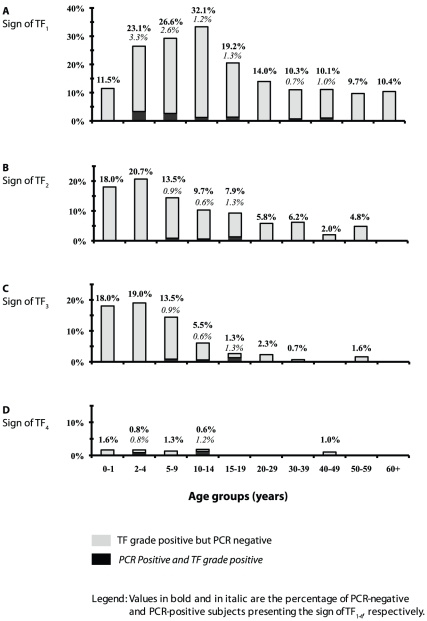
Age-specific prevalence of the left eye fine grading of TF and PCR positivity. **Fig. 4A:** Sign of TF_1_; **Fig. 4B:** Sign of TF_2_; **Fig. 4C:** Sign of TF_3_; **Fig. 4D:** Sign of TF_4_.

As result, the agreement between PCR and TF_WHO_ was poor for children of ≤9 year of age (*k* = 0.15; 95% CI: 0.01–0.25) and still poor (*k* = 0.23; 95% CI: 0.08–0.37) for older participants. Figure 4 describes the age-specific prevalence of the left eye fine grading of TF_1_ (Figure 4A), TF_2_ (Figure 4B), TF_3_ (Figure 4C) and TF_4_ (Figure 4D) versus PCR positivity.

Of particular interest were six PCR positive participants who had not have active follicular disease and were graded as TF_0_. All were female whose ages were 9, 16, 21, 57, 66 and 73 years. Two lived in houses with children who were PCR positive. Another two lived in houses in which three or more children had TF_3_. The fifth woman was aged 73 and had TI_1_ and TS_3_ with 30,918 plasmid/swab. She shared a house with two men, one aged 28 who had TF_2_ and TI_1_ and the other aged 58 with TF_1_ and TI_1_. The sixth was a 9 year-old girl with a normal exam and 37,074 plasmid/swab whose house number was missing so her household contacts could not be identified. Therefore, with the exception of the last girl, a plausible case can be made for exposure to infection and four of five had some signs of inflammation (TI of some degree).

### Organism load versus clinical sign

**Figure 5:**
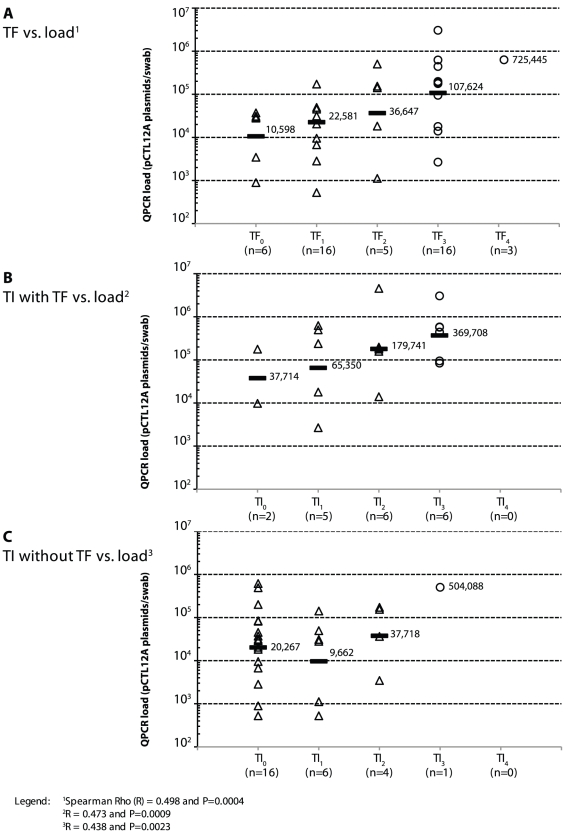
Organism load versus fine left eye clinical grading. **Fig. 5A:** TF_0–4_ vs. load^1^; **Fig. 5B:** TI_0–4_ in presence of TF_WHO_ vs. load^2^; **Fig. 5C:** TI_0–4_ in absence of TF_WHO_ vs. load^3^.

The fine grading of TF_0–4_ (Figure 5A), TI_0–4_ in presence of TF_WHO_ (Figure 5B), TI_0–4_ in absence of TF_WHO_ (Figure 5C) and TS_0–4_ in presence of TF_WHO_ was positively correlated with the organism load whereas there was no correlation for TS_0–4_ in absence of TF_WHO_ (Spearman Rho (*R*) = 0.498 and *P* = 0.0004, *R* = 0.473 and *P* = 0.0009, *R* = 0.438 and *P* = 0.0023, *R* = 0.449 and *P* = 0.0017 and *R* = −0.039 and *P* = 0.7946, respectively). The mean organism loads for the WHO grades were: TF_WHO_ present 133,252 plasmid/swab (95% CI: 2,173–8.2×10^6^), TF_WHO_ absent 26,903 plasmid/swab (95% CI: 534–1.4×10^6^), TI_WHO_ present 400,312 plasmid/swab (95% CI: 38,101–4.2×10^6^) and TI_WHO_ absent 40,135 plasmid/swab (95% CI: 655–2.5×10^6^).

### POC assay

The LPS-based POC assay yielded 18 positive individuals, 14 were PCR positive and 4 were young adults who were PCR negative and without clinical disease (TF_0_ and TI_0_). Using PCR as the comparator test, the sensitivity and specificity of the POC was 30.4% (14/46, 95% CI: 17.1–43.7) and 99.7% (1232/1236, 95% CI: 99.4–100), respectively.

## Discussion

The poor correlation between the prevalence of clinically active trachoma and evidence of infection is not new [12], [13], especially in low prevalence communities [5]. As with any infectious disease, there is an initial incubation period (4–8 days) between inoculation and the development of clinical disease [5]. This is followed by frank disease when both bacterium and clinical signs co-exist, and a later stage when the infection is no longer present or cannot be detected by diagnostic tests, yet the clinical signs persist as disease slowly resolve [5], [26]. In humans, the lag period between the last detectable bacterial shedding and the resolution of the active disease may take up to 9 months or so [5], [27]. As previously observed [7], the prevalence of active trachoma varies when one or both eyes are considered, in this study from 8.6% to 10.5%, respectively [7], [28]. For practical and economic reasons, swabs for PCR, LPS-based and 16S-RNA testing, and photographs were only collected from the left eye. Therefore, clinical/laboratory diagnostic comparisons were made only for the left eye using the consensus grading based on clinical and photographic data.

To reduce the likelihood of over-grading of clinical disease, the assessment was made both in the field using frequent reference to the WHO grading card and by independent photo-grading. Although over-grading can still occur, this combined approach reduces the risk.

A comparison of the performance data of the LPS-based POC with an analytical sensitivity of 2,500 chlamydial elementary bodies [7] from Tanzania and the current study in Australia is interesting. Although the prevalence rates of TF_WHO_ in 1 to 9 year olds are roughly comparable; 28% and 21% respectively, the intensity of disease (the proportion of those with TF and/or TI who have TI) was nearly three times higher in Tanzania (25% compared to 9.5%) as was the mean organism load (147,267 compared to 55,585 plasmids/swab). The unequal variance t-test Satterwaite using load (*P* = 0.0057) and equal variance pooled t-test using natural log transformation of the organism load indicated (*P* = 0.0333) that organism load difference between those samples collected in Aboriginal communities and those samples collected in the Masai communities were significantly different. The lower intensity of disease in Australia is reflected in the two to three times lower rates of PCR positivity in both those with TF_WHO_ (16% in Australia and 44% in Tanzania) and those without TF_WHO_ (4.3% and 9.7%). Similarly the reduced performance of the LPS-based POC assay in Australia may in part reflect the lower organism load and in part the modification of the test. The reduced disease severity and infectious load observed in Australian Aboriginal communities may reflect the dramatic differences in medical, environment and living conditions between the Masai and Aboriginal people.

Even though the detection of infection by PCR is a poor predictor of the presence of clinical disease and equally clinical disease was poorly correlated with infection, organism load was strongly correlated with the prevalence and severity of active trachoma as graded by the finer grading scheme and that 46% of infection was found in people who did not have the WHO grade of TF but who still had some milder clinical changes (TF_1_ or TF_2_). As mentioned, organism load also correlated with the fine grading of trachoma. Similar findings come from an earlier study that used a roughly similar finer grading scheme and that used both tissue culture and direct fluorescent antibody cytology to detect infection [13]. That study also found the load of infection was higher in those with more severe disease (WHO grade TF) than in those with less severe clinical disease.

In that study with a less sensitive assessment of infection 12% of infection was in those who did not have the simplified WHO grade of TF or TI. A rapid, simple and affordable POC test capable of accurate identification of active infection would nevertheless be a useful tool in trachoma control. The 16S-RNA test, a closed-system device based on visual detection of nucleic acid on a dipstick, offers several advantages and with its inherent sensitivity and thermostability and therefore has the potential to meet this need [29], [30]. Because of the sealed containment of amplified sample a dedicated laboratory is not require. Assay reagents have been converted into thermostable formulas (stability tested at 55 °C for one month and 37 °C for 12 months, data not shown) and the test uses a simplified protocol with minimum operation and sample processing made possible by a disposable modular cartridge previously described by Lee *et al* [24]. This technology, Simple Amplification-Based Assay (SAMBA) has a substantial advantage over currently available NATs, in that it is able to provide results quickly and on-site, thereby facilitating appropriate clinical support. The simplicity of SAMBA tests will allow their use in resource-poor settings in developing countries and for near-patient testing in the developed world [24]. These features may offer advantages over the LPS-based POC, although further evaluation of the 16S-RNA Chlamydia SAMBA test in areas of varying trachoma prevalence is required to better assess its performance and cost effectiveness.

Of considerable interest is the small number of individuals who had a positive PCR in the absence of a detectable follicular response (TF_0_). Some were older people with scarred conjunctiva who may be incapable of mounting such an immune response. Others may have had a transitory infection or may have been in an incubatory phase of infection. As demonstrated in this study, a rational explanation for a possible source of infection was identified in the household in all but one case. In this case an elderly woman who was PCR positive shared a house with two other adults. A treatment program that only targeted households with children would miss treating households such as this. However, a significant problem in these communities is the frequent sharing of houses and it is not unusual for children to sleep in two or three different houses each week [5]. We were not able to track this sort of movement to identify extended families. More precise data of people's movements and behaviour would be needed to explore this in further detail.

The strengths of this study include firstly the careful documentation and quantification of the two outcomes, the presence of infection and clinical trachoma. Careful specimen collection and handling limited sampling cross-contamination critical when highly sensitive NAATs capable of detecting low level of targets are used. Poor sampling methods and risk of cross-contamination in the field have caused concern about the validity of some early NAAT findings [5], [31]. We observed no significant difference between the quantity of human genomic DNA contained in negative and positive PCR swabs that makes inadequate sample collection unlikely. The repeat negative control swabs of fomites suggest that the field precautions taken to prevent cross-contamination were successful. Further, the positioning of the samples in the test plate was verified to ensure that neither positive specimens nor positive controls were adjacent on repeat testing. Quantitative PCR was used to assess the infectious load in all positive specimens.

Secondly, this study used a fine or semi-quantitative scale to grade the clinical severity of trachoma and developed a consensus-based grade that used both clinical and photographic findings. This allowed for a much more detailed assessment of the presence of clinical signs and the recognition of less advanced disease than the more frequently used WHO simplified trachoma grading system [14]. The use of a finer grading scheme is especially important in detailed research studies looking for correlation between clinical disease, environmental or genetic risk factors and the distribution of infection in a community when attempts are made to separate those with “disease” from those who are “asymptomatic”. A finer grading scheme allows the identification of subjects with clinical signs below the threshold set by the simplified WHO grading scheme because many people with less advanced disease do not fit in the clear-cut definitions used in the WHO simplified trachoma grading system.

Finally, this study was a population-based study with high community coverage that included people of all ages.

Potential weaknesses include the inability to precisely link the exposure to infection of individual participants as in these communities children in particular may move frequently from one house to house another. In addition, there were inevitably some missing data for both people and house numbers. The study did not assess the sampling, testing, clinical-grading and photo-grading agreement between swabbers, laboratories and examiners because the same swabber, laboratory and clinical-grading or photo-grading examiners were used throughout the study. The inter-operator agreement and intra-operator reproducibility of the LPS-based test [7] and clinical- versus photo-grading agreement [15] have been described elsewhere.

However, the key messages of this study are that the use of a binary grading system like the simplified WHO grading system will miss many of the more subtle issues around the identification of chlamydial infection and clinical status [13]. Specifically, 53% of the clinical disease (TF_1_ or TF_2_) and 59% of PCR positivity occurred in people with disease less severe than the WHO grade of TF. These data suggest that detailed studies of the pathogenesis, distribution and natural history of trachoma should make use of both a finer grading scheme and also quantify the infectious load. The fine grading also revealed a high proportion of people with clinical disease who were below the WHO threshold for TF. Most of the active trachoma was seen in the younger children who also had the more severe disease. Both the severity and prevalence of TF decreased with age. However, the finding of a significant proportion of people with few follicles, TF_1_ is noteworthy. Although some of this will reflect the waxing and waning of active trachoma, it suggests that in some people at least a few follicles, once formed, may persist for a long period of time or indefinitely. It is unclear whether these “persistent” follicles reflect occasional exposure to chlamydial antigens or infection or if they are a permanent tissue change.

Organism load varies in areas with different levels of endemicity and intensity of disease. In areas with a lower prevalence or intensity, laboratory tests may be of limited used for community-based assessment. However, it is in these situations that these tests would be of most use for the confirmation of sporadic cases with clinical disease. In addition, when the upper tarsal conjunctiva was swabbed transversally to collect an appropriate specimen for testing, the organism load of the consecutive swab was dramatically decreased even though the amount of collected cells was similar. This great disparity in organism load raises concerns about the use of consecutive specimens in preference to split specimens.

The second generation NAATs capable of detecting high multiple-copy of targets such as ribosomal rRNA should enhance analytical sensitivity [32]. This may enable to detect some low level infection previously missed by PCR [32], [33] or other less sensitive methods such as Real-time qPCR targeting a single copy genomic sequence such as the major outer membrane protein gene (*omp1*) or the outer membrane complex B protein gene (*omcB*) instead of the multiple-copy sequences (*Chlamydia* cryptic plasmid), LPS-based rapid test [7], culture and direct immuno-fluorescence (DFA), and so extend the period of detectable infection. A point-of-care nucleic acid amplification test based on targets with multiple copies such as 16S-RNA would be a more appropriate tool to detect low level of infection previously missed by LPS-based rapid test [7].

Finally, the current prevalence of both active trachoma and trichiasis are roughly similar to that reported 30 year ago in this region by National Trachoma Eye Health Program [15] and both of which exceed the thresholds set by WHO to define blinding trachoma as a public health problem indicate the need for appropriate interventions to control trachoma and prevent blindness in these five Aboriginal communities.

## Supporting Information

STARD flowchart STARD flowchart for reporting of studies of diagnostic accuracy.
(0.05 MB DOC)

STARD checklist STARD checklist for reporting of studies of diagnostic accuracy.
(0.05 MB DOC)
