# Supplementary material for: Correlation of Clinical Trachoma and Infection in Aboriginal Communities
Source: PLoS Negl Trop Dis. 2011 Mar 15;5(3):e986. doi: 10.1371/journal.pntd.0000986 (PMC3057949; doi:10.1371/journal.pntd.0000986)
Supplement: STARD flowchart — for reporting of studies of diagnostic accuracy. (0.05 MB DOC) [file pntd.0000986.s001.doc]

STARD checklist for reporting of studies of diagnostic accuracy

**Eligible participants**

(*n*=1545)

Unwilling to participate (*n*=222; 14.4%)

**Enrolled participants**

(*n*=1323; coverage rate = 85.2%)

**Both eyes fine clinical grading**

(*n*=1323)

**Excluded participants** (*n*=41)

- Incomplete clinical record (*n*=7)

- Unwilling to undergo swabbing (*n*=7)

- None-aboriginal participants (*n*=27)

**Left eye fine clinical grading, PCR and POC testing**

(*n*=1282)
